# A versatile acoustically active surface based on piezoelectric microstructures

**DOI:** 10.1038/s41378-022-00384-0

**Published:** 2022-05-26

**Authors:** Jinchi Han, Mayuran Saravanapavanantham, Matthew R. Chua, Jeffrey H. Lang, Vladimir Bulović

**Affiliations:** Department of Electrical Engineering and Computer Science, Massachusetts Institute of Technology, Cambridge, MA 02139 USA

**Keywords:** Electrical and electronic engineering, Sensors

## Abstract

We demonstrate a versatile acoustically active surface consisting of an ensemble of piezoelectric microstructures that are capable of radiating and sensing acoustic waves. A freestanding microstructure array embossed in a single step on a flexible piezoelectric sheet of polyvinylidene fluoride (PVDF) leads to high-quality acoustic performance, which can be tuned by the design of the embossed microstructures. The high sensitivity and large bandwidth for sound generation demonstrated by this acoustically active surface outperform previously reported thin-film loudspeakers using PVDF, PVDF copolymers, or voided charged polymers without microstructures. We further explore the directivity of this device and its use on a curved surface. In addition, high-fidelity sound perception is demonstrated by the surface, enabling its microphonic application for voice recording and speaker recognition. The versatility, high-quality acoustic performance, minimal form factor, and scalability of future production of this acoustically active surface can lead to broad industrial and commercial adoption for this technology.

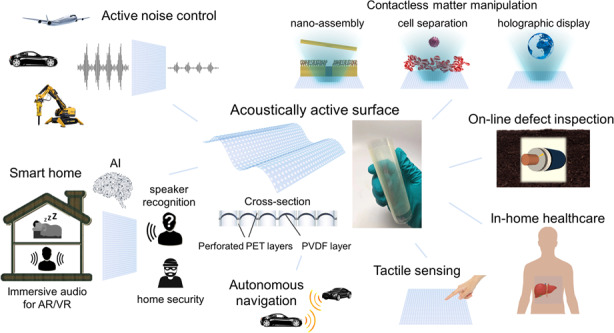

## Introduction

The fast-growing demand for acoustic transducers is motivated by diverse industrial and commercial needs, such as active noise control^1,2^, human–machine interfacing^3,4^, robotics^5^, ultrasonic imaging^6^, automated driving^7^, tactile sensing^8^, and contactless matter manipulation^9–11^, where sound may act as the medium for sensing, actuation, and communication. These technical needs are driving interest in developing low-cost and high-performance acoustic transducer technologies suitable for large-scale applications^12–20^. Among them, piezoelectric transducers are increasingly attractive due to their versatility, simple structure, low power consumption, and ease of scalability for both compact and wide-area applications^16–18^.

To address the need for large-area form factors, a variety of flexible thin-film loudspeakers have been developed based on polyvinylidene fluoride (PVDF)^1,17^, poly(vinylidene fluoride-co-trifluoroethylene) [P(VDF-TrFE)]^18–21^, piezoelectric nanoparticles^22^, voided charged polymers^23,24^, and electroactive polymers^25^. However, most designs rely on the bending of freestanding and/or curved piezoelectric layers. When they are bonded on the surface of rigid objects, the bending of the layers is greatly restricted, and degraded acoustic performance can result. This undermines the advantages of these ultrathin, lightweight and cost-effective loudspeakers and limits their application prospects. In addition, the microphonic responses of these devices, as sound receivers rather than sound generators, are often left unexplored.

**Fig. 1:**
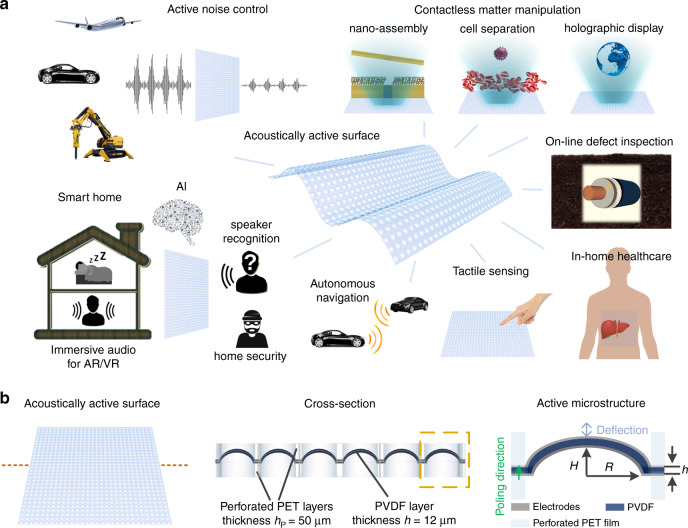
Concept and structure of the acoustically active surface. **a** Application prospects of acoustic surfaces. **b** Schematic showing the cross section of an acoustic surface and a magnified picture of an active microstructure within the array.

In the present work, we develop a large-area acoustic thin-film transducer based on an ensemble of freestanding piezoelectric microstructures that are capable of sensing and generating sound. These active acoustic surfaces are thin and flexible and can be optically transparent, allowing them to be mounted on various objects in an inconspicuous manner and thereby implemented as loudspeakers, microphones and/or ultrasonic transceivers. The freestanding protruded microstructures can vibrate freely, ensuring high sensitivity for sound generation and perception by the acoustic surface, even when it is bonded to a rigid object. The broad application scenarios represent a significant advantage over prior art involving similar acoustic films without such microstructures. Example applications of acoustically active surfaces, catering to various needs, are outlined in Fig. 1a. Our work shows that the use of densely deployed active microstructures over large acoustic surface areas brings about high-quality performance and versatility for acoustic surfaces, thus enabling a novel acoustic interface for use in artificial intelligence applications, virtual and augmented reality, robotics, smart home technologies and biomedical engineering.

## Results and discussion

### Device concept and preparation

The acoustic surface (Fig. 1b) consists of an active piezoelectric layer with a microstructure array sandwiched between two perforated polyester (PET) films. The perforated top film, which is thicker than the height of the acoustically active microstructures, protects the microstructures from collapsing under mechanical abrasion and/or impact from day-to-day human handling without affecting their vibrations. The bottom PET layer elevates the microstructures to ensure free vibration and provides a back cavity for each as well as isolation from adjoining ones. The active microstructures generate or sense acoustic waves based on the *d*_31_ piezoelectric response. The piezoelectric film is poled along the film thickness direction. When an AC voltage is applied across the piezoelectric layer, the electric field along the film thickness direction induces in-plane strain and causes the piezoelectric microstructures with clamped edges to expand and contract periodically. The microstructure vibration thereby displaces the surrounding air to generate sound. Conversely, sound incident upon the acoustic surface causes deformation of the piezoelectric microstructures and leads to charge accumulation on the electrodes, thereby transducing the incident acoustic pressure. Herein, all microstructures are connected in parallel by continuous electrode layers in order to achieve enhanced sensitivity for sound generation and perception. The microstructures can also be addressed and controlled individually or in groups by segmenting the electrodes.

**Fig. 2:**
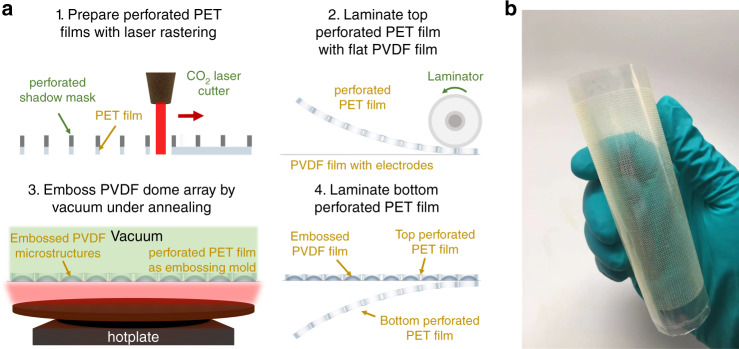
Fabrication of the acoustically active surface. **a** Process flow of device fabrication involving self-aligned, vacuum-induced embossing to create a piezoelectric microdome array. **b** A representative flexible and transparent acoustic surface. Transparent indium tin oxide electrodes are deposited on the piezoelectric layer in this example, generating a transparent, electrically active acoustic surface.

PVDF is selected for the piezoelectric layer because it is flexible, transparent and can be cost-effectively manufactured on a large scale. The microstructures in this work take the profile of spherical diaphragms, and the array of these microscale domes on the PVDF layer is prepared by a vacuum-induced self-aligned microembossing process (Fig. 2a); fabrication details can be found in the “Methods” section. A perforated PET layer is first prepared by laser rastering supplemented by a perforated shadow mask. A perforated silicon wafer is selected here as the shadow mask for ease of adapting the size and shape of through-vias in the silicon wafer by microfabrication but may not be optimal for high-throughput fabrication. The perforated PET layer is then laminated on a flat PVDF film that has electrodes on both sides. The laminate is subsequently adhered to a porous vacuum stage, and the pressure difference across the PVDF layer deforms the areas that align with through-vias in the PET film into microdomes that have the same size as the through-vias. In this method, the perforated PET layer (50 μm thick) on top of PVDF (12 μm thick) works as a mold to achieve self-aligned embossing. Finally, another perforated PET layer is laminated on the bottom side of PVDF, and the through-vias (of the same size as the domes) are manually aligned with the microdome array on PVDF. As a result of this simple self-aligned embossing, the domes do not contact the top PET layer during their vibrations. Misalignment between the through-vias in the bottom PET layer and the domes negligibly influences the vibration of these protruded microstructures (Fig. 1b) as well; such misalignment is minimized in an industrial manufacturing setting, as identical perforated top and bottom PET layers can be fabricated (e.g., by molding or casting) and easily aligned with automated systems. The proposed microembossing process is compatible with most polymer processing techniques^26^ and can be integrated with roll-to-roll processing to create microstructure arrays on PVDF films in a high-throughput manner. Here, we focus on 10 cm × 10 cm samples (yielding an active area of 9 cm × 9 cm), limited only by the toolsets available in our research setting. An example of a flexible and transparent acoustic surface is presented in Fig. 2b. Such a surface can be installed on an arbitrary object without changing its underlying appearance. For instance, the acoustic surface can be adhered to the exterior of a glass mug, augmenting it to have the functionality of a loudspeaker (Supplementary video S1).

### Tunable acoustic performance by designing active microstructures

**Fig. 3:**
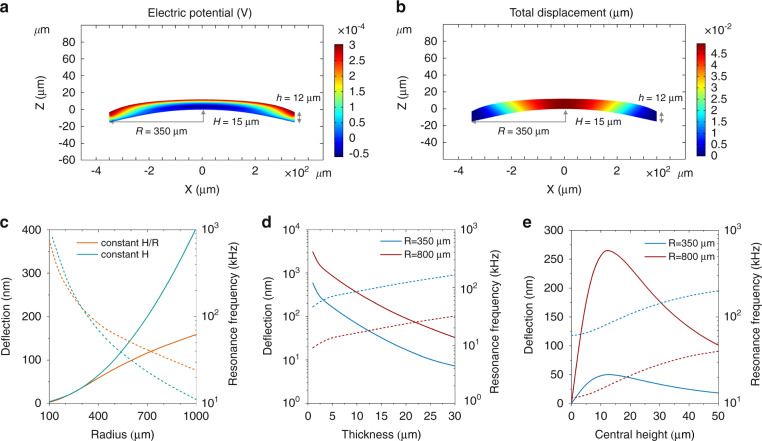
Vibration of individual piezoelectric domes and tunability enabled by the dome design. **a** Electric potential distribution induced by uniform load (1 Pa) applied on a PVDF microdome. **b** Displacement distribution over a PVDF microdome under a 10 V voltage excitation. The electrical potential in **a** and the displacement in **b** are represented by the color in each figure. The simulated dome in **a** and **b** has a 350 μm radius, a 15 μm central height and a 12 μm film thickness. **c–e** Dependence of the dome deflection (solid curves) and the resonance frequency (dashed curves) on the dome radius *R* (**c**), film thickness *h* (**d**), and central dome height *H* (**e**). The deflections correspond to 10 V applied across the electrodes. Simulation in **c** either maintains a constant aspect ratio (*H*/*R* = 5%) or a constant central height (*H* = 15 μm) while scaling up the dome size. The simulation in **d** sets a constant central height of 15 μm, while that in **e** sets a constant film thickness of 12 μm. The PVDF used in the simulation is uniaxial with *d*_31_ = 22 pC/N.

The existence of freestanding piezoelectric microdomes enables sound generation and perception even when the acoustic surface is bonded to a rigid object. Figure 3a and b present a finite-element analysis of the electric potential distribution under a uniform load (microphonic application) and the displacement distribution under an electric-field excitation (loudspeaker application), respectively, over a microdome with clamped peripheries. The profile and dimensions of a microdome play a significant role in its deflection under fixed excitation, and the deflection further determines the overall static and dynamic performance of the acoustic surface. As a result, designing these basic functional units enables characteristic tuning for the acoustic surface over a wide range. Figure 3c–e describe the dependence of the deflection at the dome center and its resonance frequency on the dome radius, film thickness, and central dome height, respectively. The design of these dome dimensions allows tailoring of the sensitivity and bandwidth for sound generation. The simulation indicates that a larger dome with reduced thickness in general exhibits more deflection and thereby a higher sensitivity. Prebending a circular thin plate to some extent is favorable for its deflection under the same excitation. This results in an optimal dome height related to its bending stiffness. Since the bending stiffness depends on the film thickness but not on the dome radius, the optimal dome height should depend only on the film thickness. Figure 3e shows that the optimal central dome heights, *H*, for radii *R* = 350 μm and *R* = 800 μm are both approximately *H* = 12 μm when the film thickness is *h* = 12 μm. Additional simulation results (Figs. S1a and S2a) verify that such an optimal height is indeed only dependent on the film thickness. The variation in the maximum deflection during scaling of the dome radius and the film thickness based on the optimal central heights can be found in Fig. S1b and S2b. The resonance frequency of a dome-shaped transducer monotonically increases with decreasing dome radius and increasing central height and film thickness. Such numerical insight can guide the design of piezoelectric microstructures for desired bandwidth or enhanced performance at a particular frequency.

### Characteristics of sound generation

**Fig. 4:**
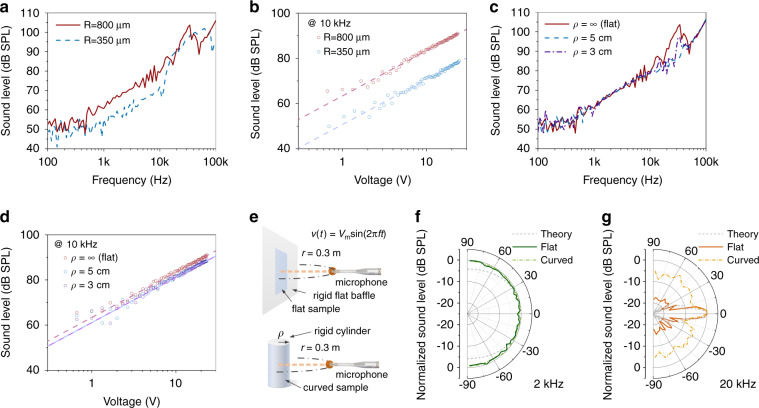
Sound generation characteristics of the acoustically active surface. **a** Frequency responses of representative acoustic surfaces. Two 10 cm × 10 cm samples (active area: 9 cm × 9 cm) with the same film thickness of 12 μm but different dome radii are compared in the figure. The sound pressure level is measured at a distance of 30 cm and a constant 10 V amplitude of the applied sinusoidal driving voltage. The pitches of the microdome array for the 350-μm-radius (*R* = 350 μm) sample and the 800-μm-radius (*R* = 800 μm) sample are 1 and 2 mm, respectively. **b** Sound pressure levels produced by the samples in response to varying drive voltages (RMS) at *f* = 10 kHz. **c** Frequency responses of an acoustic surface fixed on a rigid flat baffle or curved cylinders of different radii *ρ* under the same test conditions as (**a**). The dome radius is *R* = 800 μm, and the pitch is 2 mm. **d** Sound pressure level produced by the sample fixed on different surfaces in response to varying drive voltage (RMS) at *f* = 10 kHz. **e** Schematic showing the measurement of the directivity patterns of an acoustic surface mounted on a rigid flat baffle and an acoustic surface mounted on a rigid cylinder. **f**, **g** Directivity patterns of a representative acoustic surface (active area: 9 cm × 9 cm) on a rigid baffle (solid curves) and a representative acoustic surface (active area: 9 cm × 9 cm) on a glass cylinder of radius *ρ* = 5 cm (dotted-dashed curves) at *f* = 2 kHz (**f**) and *f* = 20 kHz (**g**), respectively, in terms of normalized SPL. The SPL at different angles to the acoustic surface is measured while maintaining a constant distance of 30 cm and normalized based on the maximum value in space. The dashed curves provide the theoretical directivities of a flat acoustic surface at 2 and 20 kHz, predicted based on the Rayleigh integral.

Sound generation of the acoustic surface is measured in an anechoic chamber setup (Fig. S3a). The frequency response of the acoustic surface acting as a loudspeaker is evaluated based on the sound pressure level (SPL) measured 30 cm away from the device under a sinusoidal drive voltage with an amplitude of 10 V and a varying frequency. The results from two acoustic surface samples based on microdomes of different sizes are compared in Fig. 4a. Although the samples are bonded on a rigid flat baffle during measurement, the freestanding microdome array enables high-quality acoustic performance in both audible and ultrasonic frequency ranges. Specifically, 61 dB SPL, 79 dB SPL and 106 dB SPL are produced at driving frequencies of *f* = 1 kHz, 10 kHz and 100 kHz, respectively, and a distance of 30 cm by the sample having 800-μm-radius microdomes driven by a sinusoidal voltage with 10 V amplitude. The corresponding sensitivity for sound generation outperforms those from large-area thin-film loudspeakers based on piezoelectric ceramic nanoparticles^22^ and cellular electrets^23,24^, which typically have much stronger piezoelectric responses than PVDF. Moreover, sound generation based on actuation of the microstructures instead of the entire film allows a large bandwidth and a high sensitivity in the ultrasonic range as well, which is superior to those of freestanding curved PVDF and P(VDF-TrFE) loudspeakers^17,19,20^. A more detailed comparison of acoustic performance between the proposed device and representative large-area thin-film loudspeakers is provided in Table 1. Our acoustic surface exhibits the highest performance for sound generation in terms of the normalized sensitivity and bandwidth. In addition, the two samples with different dome radii have different resonance peaks in Fig. 4a. The resonance frequency decreases as a result of the increased dome radius, which agrees with the trend simulated in Fig. 3c. The sound generation in response to a varying voltage is further evaluated. Example characteristics at *f* = 10 kHz are presented in Fig. 4b, which shows good linearity of sound generation of the acoustic surface. Increasing the dome size results in a larger deflection (Fig. 3c) and thereby a higher sensitivity for sound generation, as shown in Fig. 4b. Differences in the resonance frequency and the sensitivity of the two samples collectively verify the tunable characteristics of the acoustic surface afforded by designing the dome dimensions.

**Table 1:** Comparison with state-of-the-art thin-film loudspeakers

Design	Material	Active area (cm^2^)	Distance (cm)	Voltage (V_rms_)	SPL @ 1 kHz (dB SPL)	Sensitivity (mPa/V cm^2^)	Bandwidth (kHz)
This work	PVDF	9 × 9	30	7.07	61	0.039	>100
Ref. ^17^	PVDF	21 × 30	100	20	70	0.017	7
Ref. ^19^	P(VDF-TrFE)	10 × 6	5	35.4	63	0.0022	13
Ref. ^20^	P(VDF-TrFE)	10 × 6	100	17.7	55	0.035	8
Ref. ^22^	PZT/GNP	20 × 17	30	135	72	0.0017	>20
Ref. ^23^	COC	12 × 12	100	70.7	55	0.0037	>20
Ref. ^24^	COP	15 × 15	50	56.5	18	0.000021	>20
Ref. ^25^	EAP	*π* × (10/2)^2^	100	283	75	0.017	1

For sensitivity comparison, the reported SPLs at 1 kHz are converted into acoustic pressure under a 1 V_rms_ drive voltage for loudspeakers of unit area (1 cm^2^) at 30 cm away. The normalization is conducted assuming a far-field response of linear loudspeakers, i.e., acoustic pressure proportional to (active area × voltage)/distance. The bandwidths are estimated from the roll-off trend of the reported frequency responses of these devices.

*PVDF* polyvinylidene fluoride, *[P(VDF-TrFE)]* poly(vinylidene fluoride-co-trifluoroethylene), *COC* cyclic-olefin copolymers, *COP* cyclic-olefin polymers, *PZT* lead zirconate titanate, *GNP* graphene nanoplatelets, *EAP* elastomer electroactive polymer.

To demonstrate the capability of our device working on a curved object, the acoustic surface with domes of 800 μm radius is peeled from the flat baffle and then bonded on glass cylinders of different radii (*ρ* = 3 or 5 cm) in sequence for the same acoustic measurements as in Fig. 4a, b. No obvious degradation in the frequency response or the linearity of the device is observed from Fig. 4c, d. This confirms that our acoustic surface is able to achieve high-quality sound generation while mounted on curved objects.

The sound generation directivity of the device is characterized by measuring the SPL at a fixed distance (30 cm) and varying the angle away from the device normal (Fig. 4e). The directivities at *f* = 2 kHz and *f* = 20 kHz are plotted in Fig. 4f and g, respectively. The radiation patterns of the acoustic surface mounted on a rigid flat baffle (solid curves) match well with the theoretical patterns (dashed curves) predicted based on the Rayleigh integral (see details in the Supplementary Information). The result at *f* = 2 kHz (Fig. 4f) exhibits uniform sound radiation in space, as the size of the acoustic surface is smaller than the acoustic wavelength. Because the acoustic wavelength decreases at a higher frequency, the interference of acoustic waves radiated by the piezoelectric microdomes at different spots results in increased directionality (Fig. 4g) of the flat acoustic surface. Furthermore, an acoustic beam can be formed and steered in the audible frequency range when the size of the acoustic surface is made larger and the microdomes are controlled individually or in blocks as a phased array. The directivities of an acoustic surface fixed on a glass cylinder of a 5 cm radius at 2 and 20 kHz are also measured and shown as dotted-dashed curves in Fig. 4f and g, respectively. Similar to the flat acoustic surface, the sound radiation by the device bonded on a curved surface is also uniform at 2 kHz. At 20 kHz, the sound radiation becomes more uniform in space than the acoustic surface bonded on a flat baffle, as not all piezoelectric domes are facing along the same direction. The interference of sound radiation from two identical acoustic surfaces mounted on a rigid flat baffle has also been studied and is shown in Fig. S8. Enhancements of 13 dB SPL and 8 dB SPL at 2 and 20 kHz, respectively, are obtained by driving the two devices in phase compared to driving them out of phase.

### Characteristics of sound perception

Sound incident on the microdomes causes charge accumulation across the electrodes, which can be amplified and correlated to the acoustic pressure. This enables microphonic application of the acoustic surface as well. Herein, we utilize a transimpedance amplifier (Fig. S4) with a 10^8^ V/A gain and measure the microphonic response of the acoustic surface in an anechoic chamber setup (Fig. S3b). The sensitivity for sound perception is evaluated by the ratio between the amplified signal from the sample and the actual acoustic pressure, which is measured by a calibrated microphone located next to the sample.

**Fig. 5:**
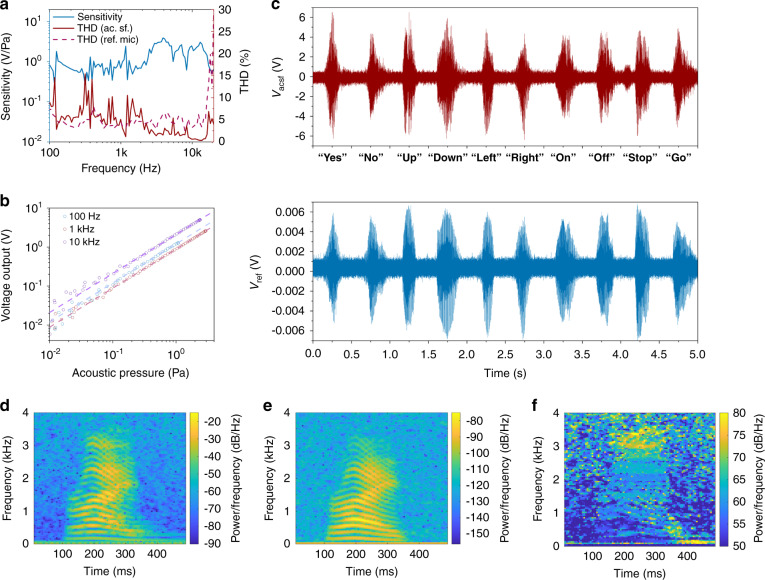
Microphonic performance of the acoustically active surface. **a** Sensitivity and THD of the acoustic surface. The THD corresponding to the reference microphone is provided as a dashed curve in the figure for comparison. A 10 cm × 10 cm sample with microdomes of radius *R* = 350 μm and thickness *h* = 12 μm is utilized. **b** Voltage output of the acoustic surface as a function of incident acoustic pressure. The output signal from the acoustic surface is amplified by a transimpedance amplifier with a 10^8^ V/A gain. **c** Comparison between the waveforms of a series of voice commands recorded by the acoustic surface and those recorded by the reference microphone. **d** Spectrogram of the waveform of voice command “RIGHT” recorded by the acoustic surface. **e** Spectrogram of the waveform of voice command “RIGHT” recorded by the reference microphone. **f** Spectrogram of the waveform recorded by the acoustic surface normalized by that recorded by the reference microphone. The voice waveforms are recorded with a 200 kS/s sampling rate. The spectrograms are plotted with a window of 5000 samples and an overlap of 4000 samples between adjoining sections. Spectrograms from another example can be found in Fig. S5.

The frequency response of a representative device is provided in Fig. 5a. The variation in sensitivity below *f* = 2 kHz is relatively small, while three resonance peaks are observed between *f* = 3 kHz and *f* = 15 kHz, well below the resonance frequency of the *R* = 350 μm domes (which the simulation predicts is at *f* = 91.7 kHz). These peaks are likely caused by a resonance of the bending mode of the entire film, which could be attenuated by improving the interfacial bonding between the piezoelectric layer and the passive PET layers. The total harmonic distortion (THD) of the acoustic surface for sound sensing is evaluated by taking the fast Fourier transform (FFT) of the amplified output signal, presented by the red solid curve in Fig. 5a. The THD of the reference microphone is obtained in the same way, which reflects distortion of the incident sound itself. The THD of the acoustic surface below *f* = 1 kHz varies between 5% and 15%, while the THD given by the reference microphone is approximately 5%. The fidelity of the acoustic surface gradually improves as the frequency increases. The THD is even smaller than that from the reference microphone above *f* = 2 kHz, owing to a better signal-to-noise ratio of the acoustic surface.

The outputs of the acoustic surface in response to varying incident acoustic pressure at *f* = 100 Hz, *f* = 1 kHz and *f* = 10 kHz are shown in Fig. 5b. These microphonic responses exhibit good linearity between 10 mPa and 3 Pa. The lower bound is limited by the signal-to-noise ratio, and the upper bound corresponds to the highest volume generated by the loudspeaker used in the experiment. Such performance indicates that the acoustic surface is able to sense and record human speech (which is typically 20 mPa, or equivalently 60 dB SPL).

To demonstrate the microphonic capability, three people are requested to read certain words and short sentences in front of the device under test. Waveforms of the output signal are acquired by an oscilloscope to record the speech and saved into different files; a detailed description of this experiment is provided in the Supplementary Information. A calibrated microphone positioned next to the acoustic surface also records the speech to provide a reference. The waveforms corresponding to a series of voice commands, recorded by the acoustic surface and the reference microphone, are compared in Fig. 5c. The spectrograms of the recorded waveforms of an example voice command can be found in Fig. 5d, e. Similar patterns are observed in both waveforms and in both spectrograms, while the acoustic surface exhibits a better signal-to-noise ratio than the reference microphone. The spectrogram of the normalized waveform, i.e., the recorded signal using the acoustic surface, normalized by that corresponding to the recording from the reference microphone, is also provided in Fig. 5f. A uniform power spectrum is observed for most of the frequency components in the colormap except for a small bright yellow region near 3.5 kHz, which coincides with the resonance peak of the frequency response in Fig. 5a. This verifies the good fidelity of the acoustic surface working as a microphone. The speech files recorded by the acoustic surface can be further utilized for speaker recognition based on a simple *k*-nearest neighbors (kNN) classifier. Such results are provided in Fig. S7 and demonstrate the prospects of the acoustic surface as a novel transducer platform for speaker recognition and human–machine interfaces.

## Conclusion

A versatile acoustically active surface is developed based on an array of piezoelectric microstructures, which act as basic units for sound generation and perception. The freestanding active microstructures can vibrate freely and thus enable high sensitivities of the acoustic surface working as a loudspeaker and/or a microphone on various objects. Such an application would be challenging for prior art that relies on bending of a large-area acoustic film and thus requires a freestanding design for the entire film. The profile and dimensions of these active structures also provide several degrees of design freedom to tune the sensitivity and bandwidth of the acoustic surface over wide ranges or to pursue enhanced performance at desired frequencies. High-quality sound generation characteristics have been achieved by the acoustic surface attributed to the active microstructures. Owing to its thin and lightweight form factor, we demonstrate that the acoustic surface can be mounted on a curved object and thereby render it acoustically active. This allows ubiquitous and nonintrusive installation of such acoustic wallpapers for enabling immersive experiences (including virtual and augmented reality, active noise control, and localized sound delivery). We also demonstrate that the acoustic surface can sense and record human voice, owing to its high sensitivity, low distortion and wide dynamic range. This enables a novel platform for applications such as smart skins for robotics and human–machine interfaces. In addition, the active microstructures can be addressed individually or in small blocks and controlled as a phased array of acoustic transducers. The resulting capability of control over standing and traveling acoustic waves along with their good performance in ultrasonic range permits an alternative acoustic system for automated vehicles^7^, volumetric displays^27^, and matter manipulation in life sciences and biomedical engineering^28^. These results collectively reflect the broad prospects of acoustic surfaces as versatile, scalable and potentially cost-effective interfaces for existing and emerging acoustic applications.

## Methods

### Device fabrication

To define the perforated PET films with high precision, a silicon shadow mask was first fabricated. Aluminum (100 nm thick) was deposited on a 6-inch silicon wafer (625 μm thick). AZ4620 photoresist was spin coated on top of the aluminum, patterned by photolithography, and developed in AZ435 before being hard baked on a 120 °C hotplate for 30 min. The aluminum layer was then wet-etched to form a hard mask underneath the photoresist soft mask. The wafer was then processed by deep reactive ion etching to form an array of through-vias. Subsequently, this silicon shadow mask was placed on top of a commercial 50-μm-thick PET film with single-side adhesive, while a CO_2_ laser was raster-scanned over the entire silicon mask. CO_2_ laser light that reaches the PET film ablates the film, forming a perforated pattern that precisely matches the through-via array pattern of the silicon shadow mask. A 12-μm-thick uniaxial PVDF film (from Poly-K Technologies, Inc.) was used as the piezoelectric sheet. The electrodes deposited on both sides of the PVDF film can be thermally evaporated silver (Ag) 50 nm thick, sputtered transparent indium tin oxide (ITO) 50 nm thick, or sputtered Ag/ITO 4 nm/35 nm thick. 50 nm-thick ITO electrodes were deposited to demonstrate an optically transparent device in Fig. 2b, and the devices used for acoustic measurements have 50 nm-thick Ag electrodes. The deposited electrodes can be segmented by using a shadow mask with desired patterns. The perforated PET film with adhesive was then laminated on the PVDF film using a laminator. The laminate was adhered to a porous vacuum stage (−25 kPa vacuum level) to emboss an array of microscale domes on the PVDF film in a self-aligned fashion. While under vacuum, the laminate was annealed at 80 °C for 5 min and then cooled down for another 5 min. This thermal treatment was repeated 3 times to ensure permanent embossing of the microstructures. A second perforated PET film with through-vias of the same size as the domes was coated with a layer of UV-curable adhesive and laminated on the backside of the microdome array. The position of the bottom perforated PET film was adjusted manually until good alignment was achieved between the through-via array of the PET film and the microdome array of the PVDF film before curing the adhesive layer under UV.

### Acoustic measurement

Both the loudspeaker response and microphonic response of the acoustic surface were measured in an anechoic chamber setup (Fig. S3). The acoustic-surface sample was bonded at the center of a 1 m × 1 m acoustic baffle, which acts as a perfect reflecting plane and reduces diffraction at the edges of the sample. In the loudspeaker measurement, a calibrated Brüel & Kjær (B&K) 4136 condenser microphone connected with a B&K 2669 preamplifier and a B&K 2822 microphone multiplexer was placed 30 cm away from the acoustic-surface sample to measure its sound generation. The sample was excited by a sinusoidal voltage signal produced by an Agilent 33522A function generator and amplified by a Crest AUDIO 1001A power amplifier. The voltage applied to the sample and the microphone output were measured by a Tektronix TDS 2014B oscilloscope. One hundred cycles of waveforms were acquired, and the amplitudes were extracted by the FFT to measure the actual drive voltage and to calculate the acoustic pressure based on the calibrated sensitivity of the reference microphone. A custom LabVIEW interface controlled the function generator for voltage sweep and the oscilloscope for data acquisition. In the microphonic measurement, a commercial loudspeaker was placed 50 cm away from the acoustic-surface sample in the chamber to generate sound of sinusoidal waveforms, and the reference microphone was positioned next to the sample on the baffle. The electrodes of the sample were connected to a transimpedance amplifier (Fig. S4). Both the output signal of the sample and that of the reference microphone were measured by an oscilloscope. One hundred cycles of waveforms were acquired, and their amplitudes were extracted by the FFT for a good signal-to-noise ratio. The custom LabVIEW interface controlled the oscilloscope for data acquisition and the loudspeaker to generate sound with sweeping frequency and volume during measurement.

## Supplementary information

Supplementary Information

Supplementary video

## Data Availability

The data presented in this paper are available from the corresponding authors upon reasonable request.
