# Supplementary material for: A versatile acoustically active surface based on piezoelectric microstructures
Source: Microsyst Nanoeng. 2022 May 26;8:55. doi: 10.1038/s41378-022-00384-0 (PMC9135689; doi:10.1038/s41378-022-00384-0)
Supplement: Supplementary file 1 — Supplementary Information [file 41378_2022_384_MOESM1_ESM.docx]

Supplementary Information

A versatile acoustically active surface based on piezoelectric microstructures

Jinchi Han^*^, Mayuran Saravanapavanantham, Matthew R. Chua, Jeffrey H. Lang^*^, Vladimir Bulović^*^

Department of Electrical Engineering and Computer Science, Massachusetts Institute of Technology, Cambridge, MA 02139, USA.

^*^Corresponding authors: hanjc@mit.edu, lang@mit.edu, bulovic@mit.edu

Contents

**Supplementary Text 1−2**

**Figure S1 to S8**

**Supplementary Text 1: Speaker Recognition**

# *Speech recording*

# The human voice collection was conducted in the anechoic chamber. The sample was mounted at the center of a baffle with a reference microphone positioned next to it. The electrodes of the sample were connected to a transimpedance amplifier (Fig. S4). The amplified output voltage of the sample and the reference microphone output were both acquired by a Tektronix DPO7254 oscilloscope. Three candidates were asked to read the same words and short sentences in front of the sample. The speeches were sampled at a 200 kS/s rate and a 400k record length and saved into different files (23 in total for each speaker).

# *Speech feature extraction*

# The speech features used for speaker recognition based on machine learning approaches are the pitch of the speech and the mel-frequency cepstral coefficients (MFCC). The dynamic speech signals are assumed to be stationary on short time scales. Therefore, the speech files were separated into short-time 25 ms intervals with an overlap of 20 ms for feature extraction. The pitch and MFCC for each frame were extracted using MATLAB built-in functions. In order to distinguish between silence and speech, the short-term power for each frame of a speech file was calculated, and their average was designated as the threshold to filter out frames that correspond to silence.

# *Training a classifier*

# After extracting features of speech files from different speakers, *k*-nearest neighbors (kNN) was used to train a classifier for speaker identification. To prepare training and test datasets, 3 speech files were randomly selected out of the 23 files recorded for each speaker and used as the test dataset, while the rest became the training dataset. Segmenting each speech file into short-time frames with an overlap produced enough data for training a classifier. The kNN classifier in Fig. S7 was trained based on 9 nearest neighbors, Euclidean distance between neighbors, and 5-fold stratified cross validation. Fig. S6 presents a comparison, in terms of the validation accuracy and the test accuracy, between using Euclidean distance and cosine distance with different numbers of neighbors on the same training dataset and test dataset. Euclidean distance with *k* = 9 exhibits the best overall performance when considering the validation accuracy and generalization.

# *Identifying speakers*

# The trained kNN classifier was utilized to label the speaker for each segmented frame of the test dataset. The results for test accuracy per frame characterized the percentage of correctly classified speech frames from each speaker and were plotted as a confusion matrix. The most likely class for all the frames of each speech file was identified as the owner of the speech file. The accuracy of speaker identification for each test speech file was also plotted as a confusion matrix. The same machine learning approach was conducted on different partitions of the datasets for training and test purpose. These results (Fig. S7) verify that our acoustic surface is able to work as a suitable platform for accurate speaker recognition.

# Supplementary Text 2: Analytical Model for Sound Generation and Directionality of the Acoustic Surface

# The sound generation at a specific location and the directivity pattern in space of the acoustic surface can be evaluated by the Rayleigh integral, which is a simplified form of the Kirchhoff-Helmholtz integral, based on the amplitude and phase of vibration of the piezoelectric microdomes. Here, we consider only the free-field response; additional assumptions are made as follows.

# The thickness of the acoustic surface is small compared to the acoustic wavelength in the frequency range of interest.

# The acoustic surface is mounted on a large rigid baffle which acts as a perfect reflecting plane. Correspondingly, diffraction around the sample is neglected.

# The overall motion (e.g., bending, wrinkling) of the piezoelectric film is ignored. Only the microdomes are considered to be the active area. The flat area between adjoining domes are assumed to be perfectly clamped with zero surface velocity as a result of ideal bonding to the rigid baffle.

# The frequency of excitation is well below the resonance frequency of the microdomes (i.e., quasi-static state). As a result, the phase of dome vibration is assumed dependent only on the phase of drive voltage.

# The vibration of a microdome is not impeded by air damping and other loss mechanisms.

# The acoustic pressure *p*(r_d_) produced by the acoustic surface at a point located at r_d_ is given by

, (1)

# where r_d_ is the vector from the designated origin to the observation point; r_d_’ is the vector from the origin to the location of d*S* on the acoustic surface; *v*(r_d_^’^) is the amplitude of surface velocity at r_d_’ normal to the plane of the acoustic surface; *ρ* is the density of air; *k* is the wavenumber; *c* is the sound speed; *V*(r_d_’) is the amplitude of the drive voltage applied at r_d_’; and *φ*(r_d_’) characterizes the relative phase of surface velocity, dependent on the phase of drive voltage at r_d_’.

# For our samples, which are based on microdome arrays, the Rayleigh integral can be discretized. The acoustic pressure at position r_d_ becomes a summation of the acoustic pressure generated by individual domes at r_d_, thus

, (2)

# where r_d_*_n_*’, *A_n_*, ${\bar{\boldsymbol{w}}}_{\boldsymbol{n}}$ are the location, area and average deflection (along the normal direction) of microdome *n*, respecitvely; *f* is the frequency of excitation; *V_n_* is the amplitude of the drive voltage applied on microdome *n*; and *φ_n_* characterizes the relative phase of vibration of microdome *n*.

# The sound generation of the acoustic surface was characterized by driving all the microdomes in phase with the same voltage. The microdomes on each sample we fabricated also have identical dimensions and thereby the same area and average deflection. As a result, Equation 2 can be further reduced and utilized to calculate the directivity pattern in theory (dashed curves in Fig. 4f,g). It should be pointed out that Equation 2 is also capable of calculating sound generation and the directionality of a pixelated acoustic surface when microdomes of different sizes are driven by voltages of different amplitudes and phases.

# The acoustic pressure can be further converted into sound pressure level by

$SPL(\mathbf{r}_{\text{d}},f,V_{n})=20\log_{10}\frac{\left|p(\mathbf{r}_{\text{d}},f,V_{n})\right|}{p_{0}}$, (3)

# where *p*_0_ = 20 μPa is the reference sound pressure.

#
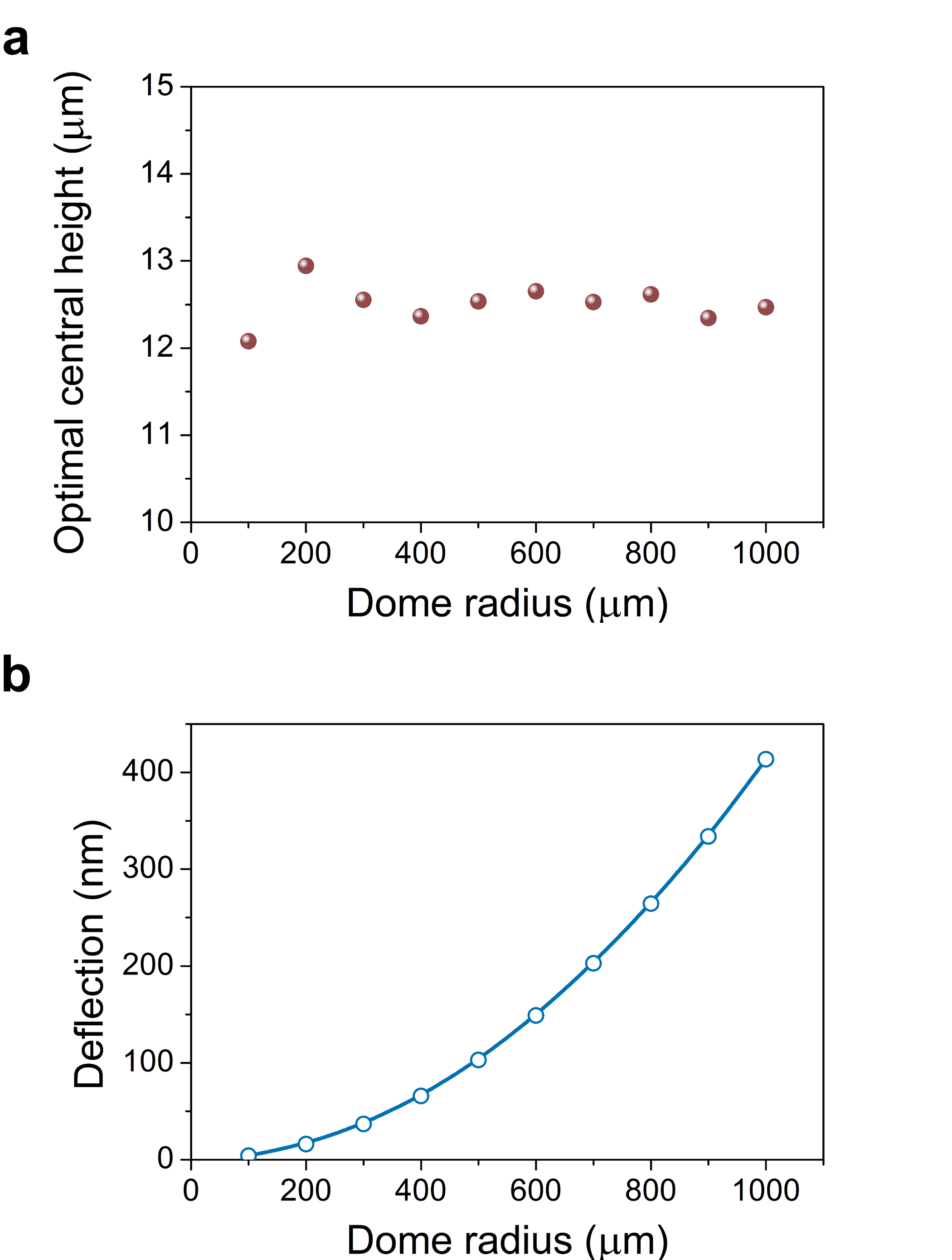


# Fig. S1. Optimal central dome height with respect to dome size scaling. a, Optimal dome heights for maximized displacement, obtained from COMSOL simulation of several microdomes constructured with different radii. The film thickness is assigned to be *h* = 12 μm for all domes. b, The maximum displacement based on the optimal central height during dome size scaling. A static 10 V voltage across the electrodes is assigned as the excitation. The piezoelectric material is uniaxial PVDF with a *d*_31_ costant of 22 pC/N.

#
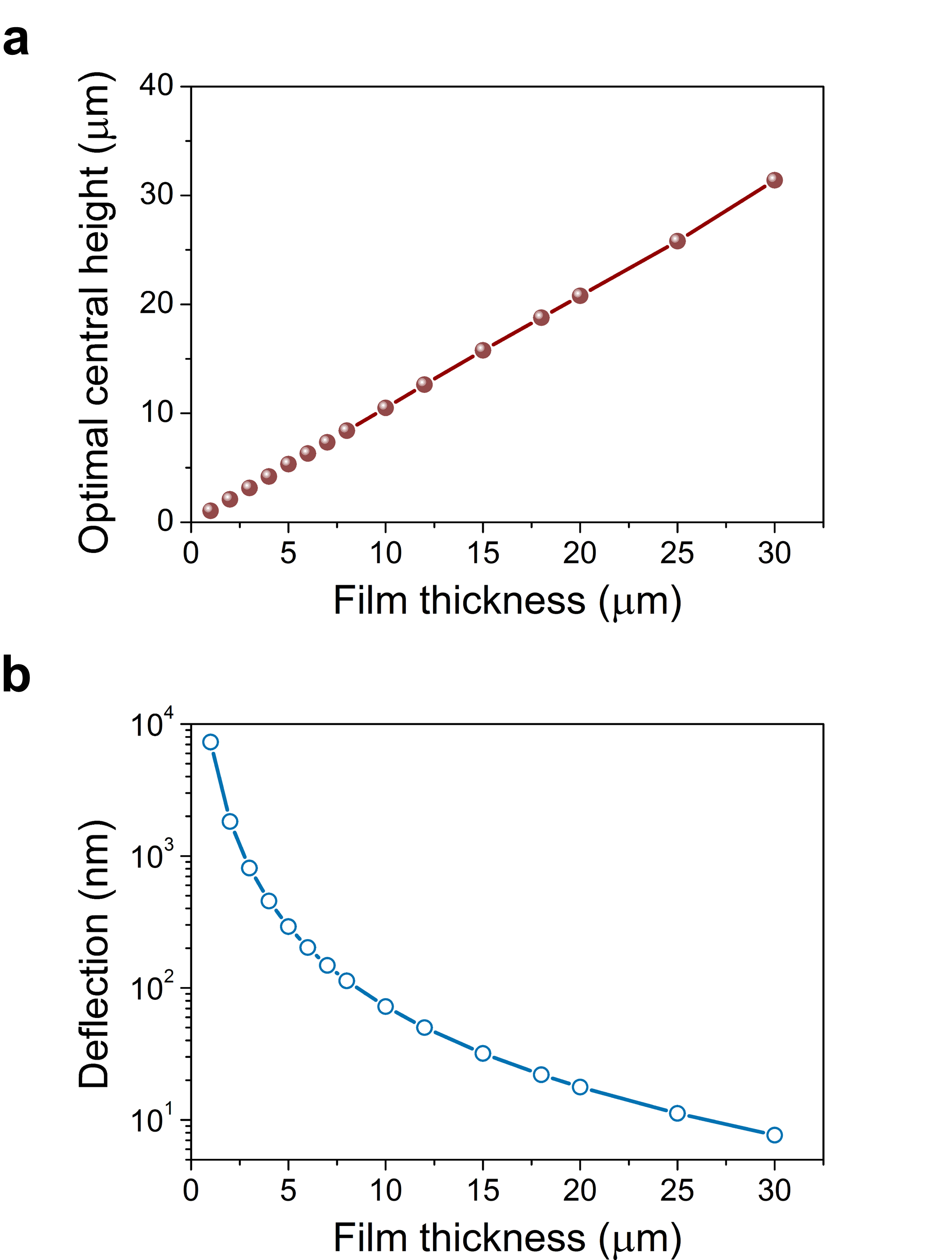


# Fig. S2. Optimal central dome height with respect to film thickness scaling. a, Optimal dome heights for maximized displacement, obtained from COMSOL simulation of several microdomes constructured with different film thicknesses. All domes have the same radius of *R* = 350 μm. b, The maximum displacement based on the optimal central height during film thickness scaling. A static 10 V voltage across the electrodes is assigned as the excitation. The piezoelectric material is uniaxial PVDF with a *d*_31_ costant of 22 pC/N.

#
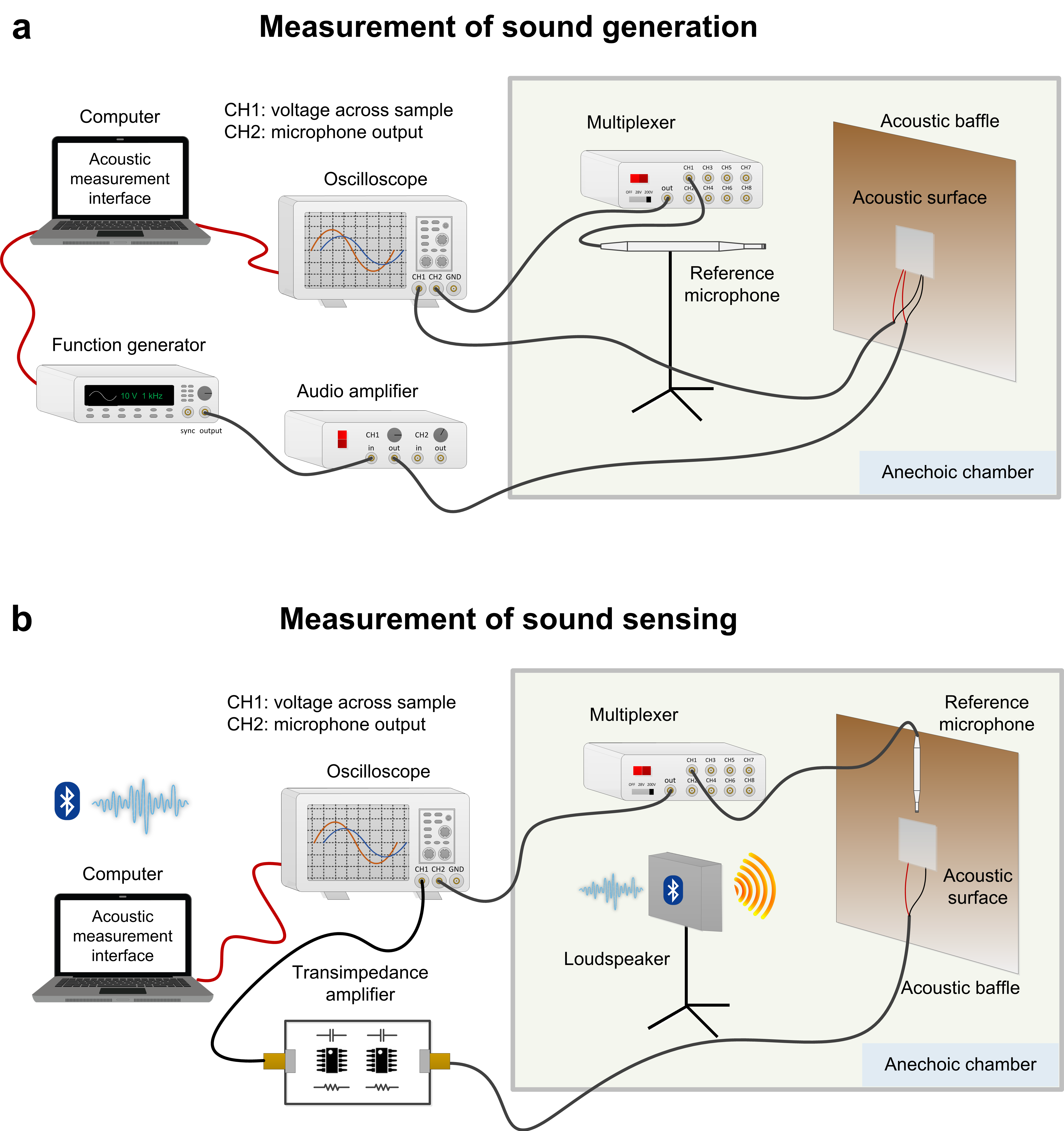


# Fig. S3. Schematic of the acoustic measurement setup in the anechoic chamber. a, Setup for the measurement of sound pressure level produced by the acoustic surface. The reference microphone is located 30 cm away and oriented along the normal direction to the sample. b, Setup for the measurement of sound sensing by the acoustic surface. The reference microphone is placed next to the sample. The sample is bonded at the center of a rigid baffle in both loudspeaker and microphonic measurements.

#
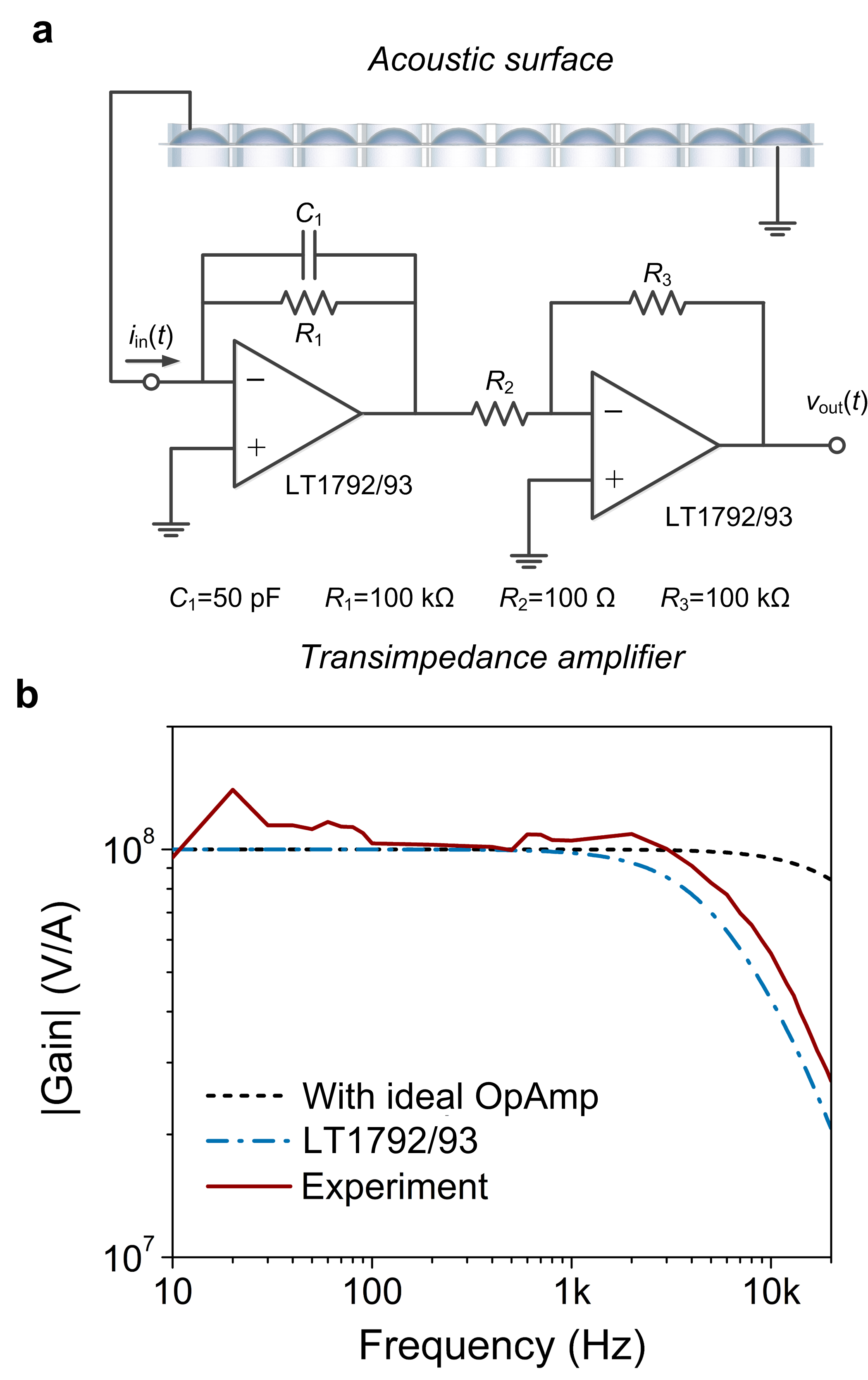


# Fig. S4. Transimpedance amplifier used for microphonic application. a, Circuit diagram of the transimpedance amplifier. Parameters of the electric components are also marked in the figure. b, Simulated and experimental frequency responses of the transimpedance amplifier in terms of the gain. The simulation was conducted using LTSpice. The dashed curve and the dotted-dashed curve present the simulation result based on an ideal operational amplifier and that based on LT1792/93 operational amplifier in the library of LTSpice, respectively. The solid curve shows the measured frequency response of the transimpedance amplifier used in all experiment.

#
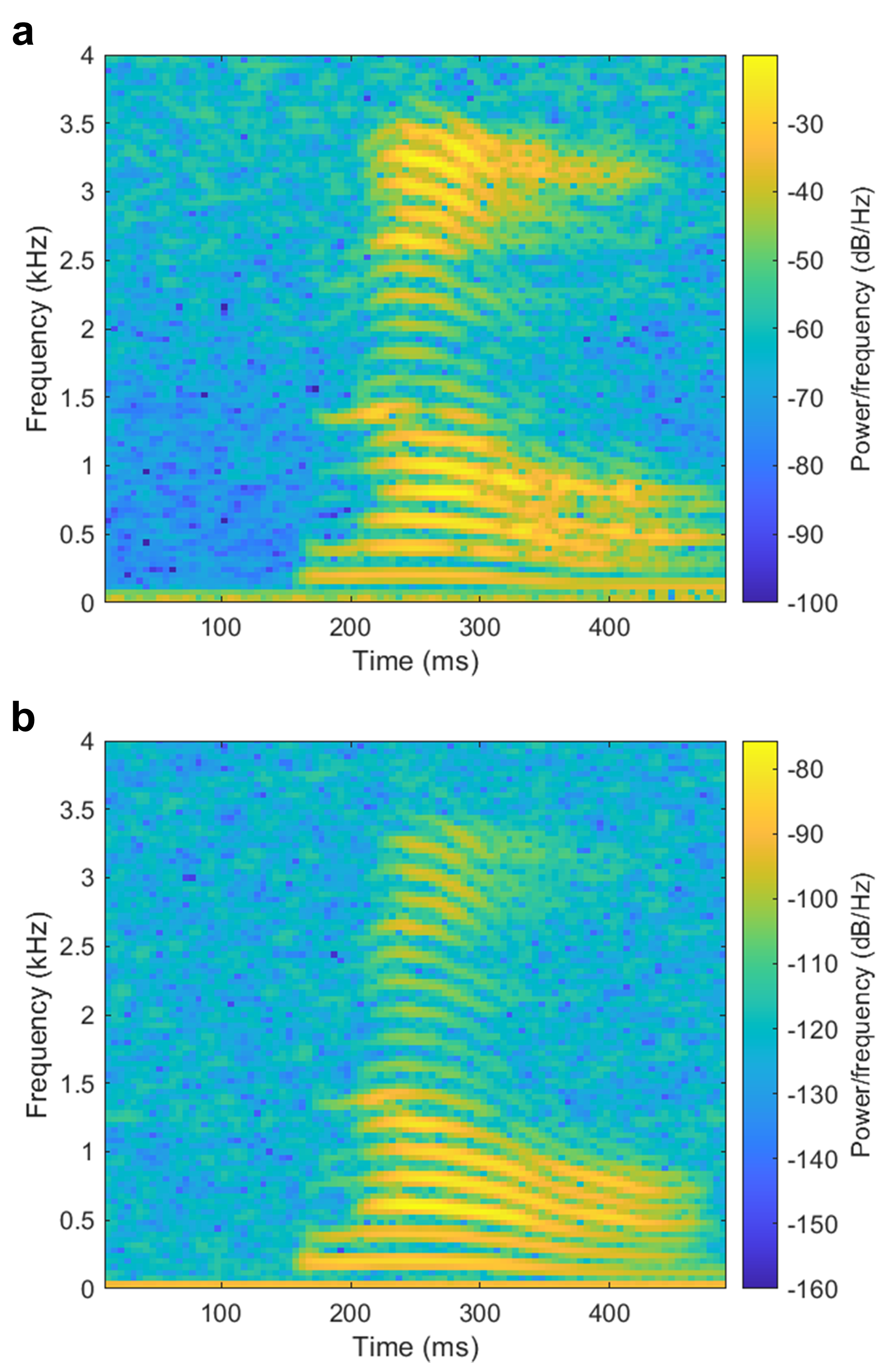


# Fig. S5. Spectrograms of the recorded waveforms of an example speech file. a, Spectrogram of the waveform of voice command "NO" recorded by the acoustic surface. b, Spectrogram of the waveform of voice command "NO" recorded by the reference microphone. The voice waveforms were recorded with a 200 kS/s sampling rate. The spectrograms were plotted with a window of 5000 samples and an overlap of 4000 samples between adjoining sections.

#
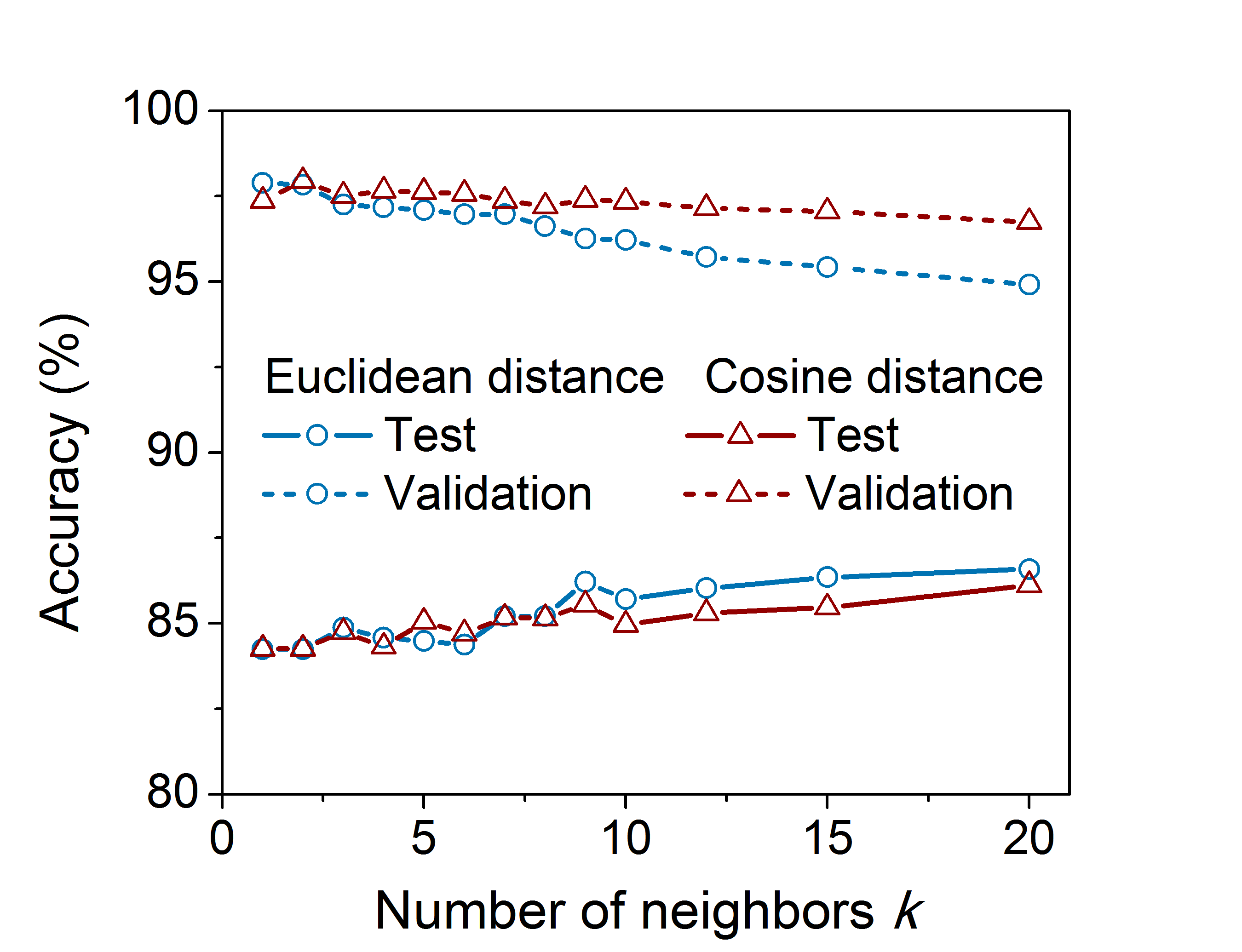


# Fig. S6. Accuracy of trained kNN classifier for speaker recognition based on different numbers of neighbors and types of distance measurement. Validation accuracy and the test accuracy per speech frame for identifying the speaker are represented by the dashed curve and the solid curve, respectively. Results based on two types of distance measurement, i.e., Euclidean distance and cosine distance, are also compared in the figure.

#
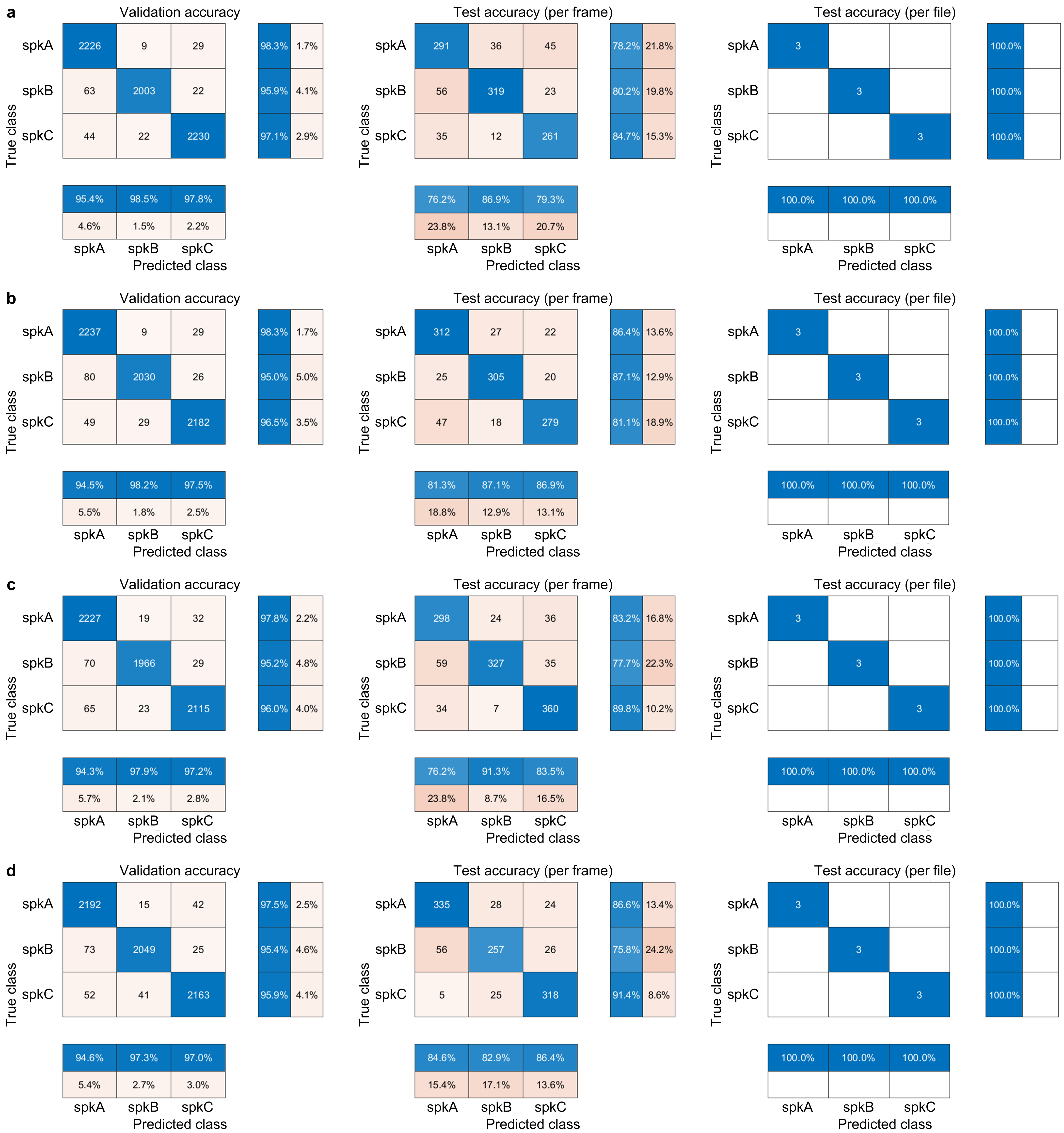


# Fig. S7. Results of speaker recognition based on different partitions of the speech files for training and test purpose. a-d, Validation accuracy per speech frame, test accuracy per speech frame and test accuracy per speech file based on four random partitions of the dataset for training and testing. Nine nearest neighbors, Euclidean distance between neighbors, and five-fold stratified cross validation are adopted for training the kNN classifier. For each partition, the trained kNN classifer achieves an overall test accuracy above 80% for each segmented speech frame and a 100% accuracy for each speech file.

#
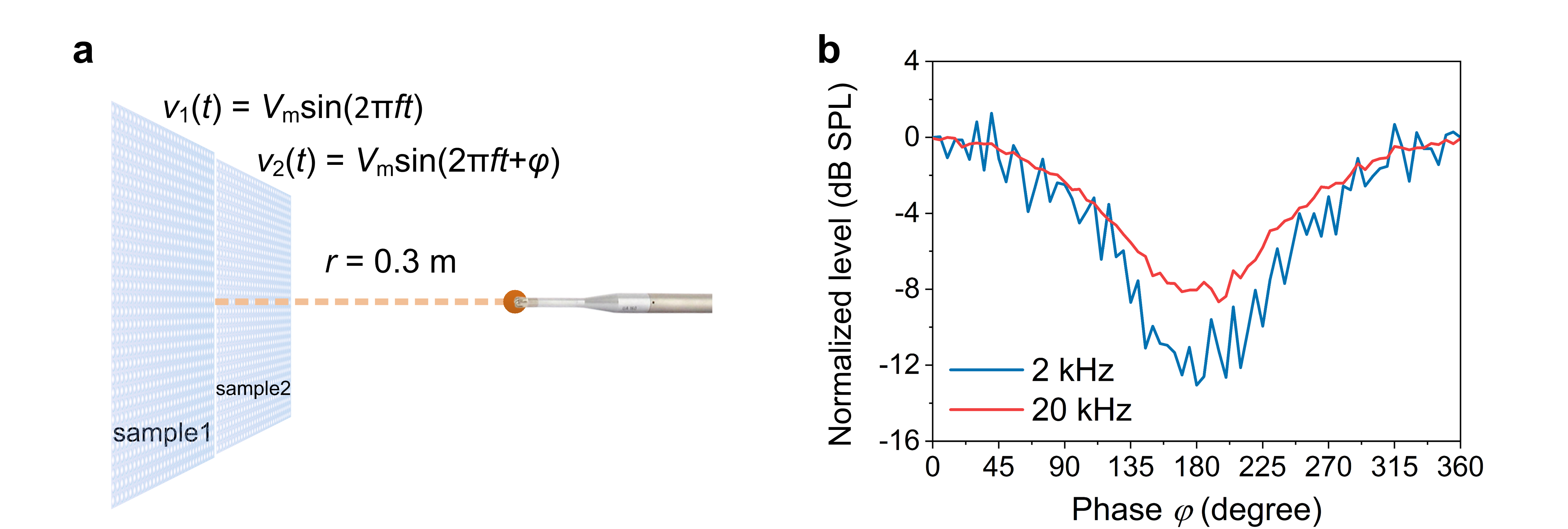


# Fig. S8. Sound generation by two acoustic surfaces driven at difference phases. a, Schematic showing the characterization of the interference of sound generated by two acoustic surfaces. Two identical samples are positioned next to each other. The microphone is located 30 cm away, oriented along the normal direction towards the middle of the two samples. b, Normalized SPL in response to varying the phase difference, *φ*, in the driving sinusoidal voltage of the two acoustic surfaces at *f* = 2 kHz and *f* = 20 kHz. The SPL is normalized by the values at *φ* = 0º.
